# The Minimum Age for Training

**Published:** 1920-10-02

**Authors:** 


### THE GENERAL NURSING COUNCIL.

### The Minimum Age for Training.

The first autumn meeting of the General Nursing Council was held on Thursday, September 23, at 2 o'clock, at the Ministry of Health. Mr. J. C. Priestley, K.C., was in the chair, and those present were Lady Hobhouse (appointed by the Privy Council), Rev. G. B. Cronshaw, Dr. E. W. Goodall, M.D., Dr. Bostock Hill, Dr. Bedford Pierce, M.D., and Sir T. Jenner Verrall, M.D. (all appointed by the Minister of Health); Miss A. Cattell, private nurse; Mr. T. Christian, Banstead Lunatic Asylum; Miss A. Coulton, Shadwell Children's Hospital; Miss Cox Davies, R.R.C., Royal Free Hospital; Miss A. Dowbiggin, C.B.E., R.R.C., Edmonton Infirmary; Mrs. Bedford Fenwick; Miss A. Lloyd Still, C.B.E., R.R.C., St. Thomas's Hospital; Miss M. MacCallum, Professional Union of Trained Nurses; Miss I. Macdonald, Royal British Nurses' Association; Miss M. E. Sparshott, C.B.E., R.R.C., Manchester Royal Infirmary; Miss Peterkin, Superintendent Q.V.J.N.I.; Miss Ellinor Smith, Welsh Superintendent; Miss E. C. Swiss, health visitor for Willesden; Miss Villiers, Stockwell Fever Hospital; Miss C. Worsley, Liverpool Children's Hospital; and Miss C. Seymour Yapp, Ashton-under-Lyne Lake Hospital (all nurses appointed by the Minister of Health). The Hon. Mrs. Eustace Hills (appointed by the Board of Education) wrote from Argyll regretting her inability to be present.

The Chairman read a letter from the Minister of Labour in relation to the inclusion of nurses in the Restriction of Hours of Employment Bill, which it is proposed to present to the House of Commons in the coming session. A draft was laid before the House last session, but was sent to the Minister of Labour for consideration. This Bill was brought before a Committee of working ladies at a Round Table Conference; it was discussed for some hours, and at the end of the discussion it was decided that the General Nursing Council be asked not to express any opinion as yet either one way or another.

It was therefore agreed by the Council that its opinion on the subject should remain in abeyance.

### The Age of Entry in Poor-Law Infirmaries.

The Chairman then read a letter from Dr. Addison, in which he stated that he would be glad if the Council would consider the minimum age of entry for probationers in Poor-Law infirmaries, as he had received requests that this might be lowered from twenty-one. The Chairman said he believed that it is usually considered to be a moral danger to allow girls to become probationers at too young an age. But it must be remembered that the average girl leaves school at seventeen or eighteen, and her parents cannot afford to keep her at home until she is twenty-one. He was recently talking to a doctor in the country, who said that as far as health goes there is no reason why a girl should not be permitted to enter at nineteen. Indeed, the Council had fixed the age for registration as early as twenty-one.

Miss Sparshott said that the question had received the attention of the Board of the Manchester Royal Infirmary, and it had been decided to fix the entrance age at nineteen, since enough good nurses could not be obtained from girls between twenty-one and twenty-three. She maintained that girls of the present day are older for their age than

**The General Nursing Council**—(*continued*).

previously, as a result of their education and home training. Their general outlook has been quite altered. Many hospitals are instituting preliminary training-schools, and these help the young nurses and make their work on entry into the wards less hard.

Miss MacCallum asked if the age at which a nurse might enter a hospital did not depend upon the hours she had to work.

Mrs. Bedford Fenwick (who remarked that she was glad to see the Press present) said that, as far as moral danger was concerned, she could imagine no place where a young woman would be better looked ofter than a training-school, where she is under careful supervision. She was in favour of reducing the age minimum, because the hospitals wanted the pick of the basket, not the leavings. Many girls leave school even earlier than eighteen, and get absolutely absorbed into other professions. She wished to propose that the Council recommend that the age at which a nurse may be admitted into a Poor-Law infirmary be nineteen.

Miss Macdonald, in seconding the motion, said that she had had a good deal of experience in the hygiene and psychology of the adolescent girl, and could find no reason why nineteen should be too young for probationership.

Miss Dowbiggin considered that at nineteen a nurse was easier to teach and more adaptable to rules than at twenty-one, and absorbed her training better.

Mr. Christian submitted that it was most important to consider the number of hours that the nurse will have to work if she is to be admitted under twenty-one. He desired to add an amendment to this effect. He also hoped that there was no idea of employing cheaper labour because the candidates are young. He thought it would be a great mistake if the mental hospitals were made to receive nurses under twenty-one; the work was not suitable.

Sir Jenner Verrall observed that because Poor-Law infirmaries were permitted to receive girls at nineteen, there would be no obligation for them or for any other institution to do so, and it would be entirely in the hands of the individual hospital. Personally, he thought that the girl who has recently left school finds it much easier to assimilate her training, as she has not forgotten how to learn.

Dr. Goodall said that at many fever hospitals probationers are taken at nineteen, or even eighteen, because they go on afterwards for general training.

Mrs. Bedford Fenwick asked if the matter affected the voluntary hospitals at all.

The Chairman explained that only the Poor-Law infirmaries were involved, they alone had to ask the permission of the Ministry before reducing their age minimum.

Dr. Bedford Pierce said that it was absurd to stipulate that the age should be dependent on the number of hours of work. They might as well make rules about the food and sleep of the nurses.

The following amendment was then put to the meeting: That the age of the probationers only be lowered to nineteen for entry to Poor-Law infirmaries, when the hours of work do not exceed ninety-six a fortnight. Proposed by Mr. Christian, seconded by Miss MacCallum.

The amendment was lost by three votes to seventeen.

Lady Hobhouse remarked that the amendment included the question of working hours, which had already been discussed by the Council.

Miss Cox Davies said that it was for that reason she had refrained from voting.

The Chairman said the two questions were closely allied, and as the consideration of the number of hours of work had been postponed until the next meeting, he had hoped the amendment would not be pressed.

Dr. Goodall asked if there was anything in the nursing at Poor-Law infirmaries which made it necessary that nurses should be admitted at an earlier age than elsewhere.

Miss Dowbiggin replied that it gave the infirmaries a wider choice of candidates.

The resolution was then put to the meeting, and carried by seventeen votes to one.

### The Problem of the Small Hospitals.

The Chairman read a letter from the Board of the Hospital at Stratford-on-Avon. He had received similar letters from Aberystwyth Infirmary, Denbigh Infirmary, Bideford Hospital, Louth Hospital, Wolverhampton Hospital for Women, Scarborough Hospital, Redditch Hospital, Monmouth Hospital, and Tyrrell Hospital, Ilfracombe. The letters referred to a resolution passed at the Nursing and Midwifery Conference held on June 23 last as follows:—

"That this meeting desires to call the attention of the General Nursing Council to the present unsatisfactory condition of the small and special hospitals which cannot offer a full certificate to their probationers, and respectfully urges the Council to consider a scheme for affiliation between such hospitals and the larger schools."

The letter from Stratford pointed out that the small hospitals have no inducement to offer their probationers, since there is no reduction for the training given by them when the girls pass on to the larger schools. With the passing of the Registration Bill the supply of nurses is still further diminished. It was also pointed out how difficult it is to obtain trained sisters in institutions where there is no adequate supply of probationers, and thus the patients are penalised. The Hospital Board requested that the Council might consider a scheme for reduced training in large hospitals for the probationers from the small institutions, and a recognised certificate of affiliation.

The Chairman said that he would suggest the matter be referred to the Examination and Education Committee, as the question did not apply to existing nurses, and that the Registrar be asked to write to the hospitals who had written and inform them that this course would be adopted. This was agreed to by all.

### V.A.D.s and State Registration.

The Chairman then read a letter from the Joint V.A.D Committee, in which it was requested that the Council would inform the Committee whether the V.A.D. members who had served for three years during the war in a military hospital would be eligible for State Registration. Mr. Priestley added that some V.A.D.s had approached him to know if nothing could be done to reduce the period of general training for those who had three years' experience and teaching, if any, during the war.

Miss Sparshott pointed out that many hospitals have reduced four years' training to three for certain specified V.A.D.s.

Miss Seymour Yapp said that at Ashton-under-Lyne the V.A.D.s who had worked in the military ward were in some cases later received as second-year nurses in the civilian wards.

Miss Cox Davies begged to move that the answer to the letter might be in the direct negative. She was seconded by Miss Cattell.

Mrs. Bedford Fenwick asked if it was proposed that the V.A.D.s should be included on the main register or the men's register.

The Chairman said that apparently V.A.D.s did not come within any of the qualifications which had been laid down for admittance to the Register.

The Resolution that the answer must be in the negative was passed unanimously.

### The Rules for Registration.

The Rules for Registration were then carefully discussed and gone into from every point of view. The matters for debate included the qualifications necessary for admittance to the different parts of the Register and the framing of the rules, the problem of nurses from women's and special hospitals, the date of the publication of the Register, the fee for registration of a Scottish or Irish nurse on the English Roll, and several minor points. Every difficulty was threshed out thoroughly, the meeting lasting over four hours. The Rules, which are not yet public, are now ready to be re-drafted for the last time, and will then be presented to the Minister of Health for approval.

The next meeting was fixed for Friday, October 15.

